# Uncertainty Modeling of a Modified SEIR Epidemic Model for COVID-19

**DOI:** 10.3390/biology11081157

**Published:** 2022-08-02

**Authors:** Yanjin Wang, Pei Wang, Shudao Zhang, Hao Pan

**Affiliations:** Institute of Applied Physics and Computational Mathematics, No. 2, Fenghao Donglu, District Haidian, Beijing 100094, China; wangpei@iapcm.ac.cn (P.W.); zhang_shudao@iapcm.ac.cn (S.Z.); pan_hao@iapcm.ac.cn (H.P.)

**Keywords:** COVID-19, mathematical epidemic model, SEIR, uncertainty quantification

## Abstract

**Simple Summary:**

This paper proposes a modified SEIR model to study COVID-19 in Wuhan. The modified model is calibrated by the public number of COVID-19 hospitalization cases in Wuhan. The paper further uses this model to estimate the earliest date of COVID-19 infection in Wuhan, which is in agreement with some existing results.

**Abstract:**

Based on SEIR (susceptible–exposed–infectious–removed) epidemic model, we propose a modified epidemic mathematical model to describe the spread of the coronavirus disease 2019 (COVID-19) epidemic in Wuhan, China. Using public data, the uncertainty parameters of the proposed model for COVID-19 in Wuhan were calibrated. The uncertainty of the control basic reproduction number was studied with the posterior probability density function of the uncertainty model parameters. The mathematical model was used to inverse deduce the earliest start date of COVID-19 infection in Wuhan with consideration of the lack of information for the initial conditions of the model. The result of the uncertainty analysis of the model is in line with the observed data for COVID-19 in Wuhan, China. The numerical results show that the modified mathematical model could model the spread of COVID-19 epidemics.

## 1. Introduction

In December 2019, a new coronavirus, severe acute respiratory syndrome coronavirus 2 (SARS-CoV-2) was first discovered in Wuhan, Hubei Province, China. The World Health Organization named the disease caused by this coronavirus disease 2019 (COVID-19) on 11 February 2020. In January 2020, the COVID-19 epidemic began in Wuhan [1]. A serial strategy was adopted to control the COVID-19 epidemic in Wuhan. First, a lockdown of Wuhan and nearby cities in Hubei Province was enforced to prevent the further exportation of infected individuals to the rest of China [2], which was crucial to force COVID-19 transmission down under control [3]. Then, many health care teams were sent from outside of Hubei Province to support Wuhan. Anyone who went outdoors was required to wear a mask [www.chinatoday.com.cn/ctenglish/2018/ttxw/202009/t20200909_800220271.html, accessed on 9 September 2022]. In particular, clinical symptoms began to be used to confirm the diagnosis of COVID-19 in the worst-affected area of China starting on 12 February 2020, and CT imaging results were used as clinical diagnostic criteria for COVID-19, although only in Hubei Province [4]. Owing to this strategy, China gained control over the epidemic. The daily number of new confirmed cases in Wuhan dramatically dropped from the thousands to the single digits. The Chinese strategy for controlling COVID-19 was successful. Chinese scientists have also substantially contributed to the successful control of COVID-19 in Wuhan. However, the COVID-19 epidemic has spread to many other countries worldwide. Currently, scientists are investigating whether the Chinese strategy for controlling COVID-19 could work elsewhere [5]. It is necessary to use a mathematical epidemic model to study the transmission dynamics of COVID-19 epidemic in Wuhan.

It is difficult to study an epidemic partly because of the many uncertain factors at the individual, institutional, and governmental levels regarding a new and severe infectious disease [6]. Over the last few decades, mathematical models of disease transmission have been used to develop effective intervention strategies for infectious diseases and have become an increasingly powerful tool for the analysis of the epidemiological characteristics of infectious diseases [7,8,9,10,11]. The most common class of epidemiological mathematical models is the compartmental model. One of the popular compartmental approaches assumes that a susceptible individual first goes through a latent period (and is said to become exposed or in class E) after being infected and then recovers, in an approach known as the SEIR model. The SEIR model has been used to study two other coronavirus epidemics (SARS and MERS) since 2003 [6,12,13,14,15]. Additionally, some researchers have used mathematical models to study COVID-19 [16,17,18,19,20,21,22,23,24]. One study was performed to optimize the clinical diagnosis of COVID-19 [25]. Ref. [26] reconstructed the full transmission dynamic of COVID-19 in Wuhan using a modified model, which extended the classic SEIR model to include seven compartments: susceptible (S), exposed (E), pre-symptomatic infectious (P), ascertained infectious (I), unascertained infectious (A), isolation in hospital (H), and removed (R). Furthermore, ref. [27] used a coalescent framework to combine retrospective molecular clock inference with forwarding epidemiological simulations to determine how long COVID-19 could have circulated before the time of the most recent common ancestor of all sequenced COVID-19 genomes. However, the initial source of the transmission of COVID-19 remains obscure. Refs. [26,27] proposed some complex modified SEIR models to model the COVID-19 epidemic in Wuhan. The complex model may induct some additional uncertainty factors. One objective of this study was to use a simple mathematical model to reconstruct the COVID-19 transmission in Wuhan.

This study’s main objective was to use a mathematical model to study the COVID-19 epidemic in Wuhan. The mathematical model uses little information from the early period of COVID-19 in Wuhan. To this end, we first designed a modified mathematical model based on the classic SEIR model to obtain the simulation data. Then, we calibrated the parameters of the proposed model based on the simulation data as mentioned above and the real public data for the COVID-19 epidemic in Wuhan. At last, we quantified the uncertainties of the COVID-19 epidemic model in Wuhan to show the applicability of the proposed epidemic model and infer the initial data of the COVID-19 in Wuhan.

The rest of this paper is organized as follows. In Section 2, the mathematical model is described with some uncertain parameters. In Section 3, the calibration of the mathematical model using public data is shown. In Section 4, modeling of the COVID-19 epidemic in Wuhan is illustrated, which includes the control basic reproduction number and inverse deduction of the early data of COVID-19 in Wuhan. In Section 5, some conclusions are presented. 

## 2. Mathematical Epidemic Model for COVID-19 in Wuhan

The first objective of this study was to develop a mathematical model for the spread of COVID-19 in Wuhan, China, by accounting for the specific characteristics of the epidemic. Many researchers have made significant progress with the SEIR model, where S, E, I, R denote mutually exclusive classes containing susceptible, exposed (latent), infectious, and recovered individuals. Ref. [26] proposed a modified SEIR model (known as the SAPHIRE model) for COVID-19, and a coalescent framework to combine retrospective molecular clock inference with the SAPHIRE model is used in [27] to determine how long SARS-CoV-2 could have circulated before the time of the most recent common ancestor of all sequenced SARS-CoV-2 genomes. In this work, we studied the period after the lockdown of Wuhan city, when there was no inflow or outflow of people. Moreover, in this period, owing to the strategy in [4], China gained control over the COVID-19 epidemic. Thus, the compartment of unascertained infectious (A) is inapposite in this study, so it is necessary to propose a modified SEIR model. 

**Figure 1:**
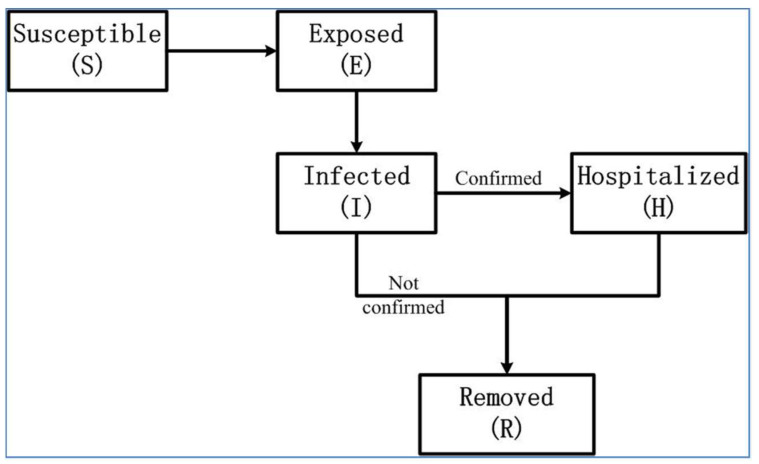
Illustration of the SEIHR model. This model includes five compartments: susceptible (S), exposed (E), infected (I), hospitalized (H), and removed (R). After becoming infectious, they progressed to two classes: hospitalized with confirmed COVID-19 patients and removed without confirmation.

As different epidemics have different transmission characteristics, using mathematical models to describe them is not straightforward. Based on the epidemiology survey, the latency period of COVID-19 is generally from 3 to 7 days, with a maximum of 14 days. We classified the population into five compartments (Figure 1). Our model also considered that variations in transmission affected the daily rate of change from the susceptible class to the exposed class. We divided the population into susceptible (S), exposed (E), infected (I), hospitalized (H), and removed (R). Thus, based on the SEIR model, we propose a modified SEIR model that models the movement of people within five states, S, E, I, H, and R, where the infected class, I, includes individuals with symptomatic and asymptomatic infections, and the removed class, R, includes recovered and deceased individuals without considering natural births and deaths. In our model, we introduced compartment H because the number of the daily hospitalized patients can be obtained from the public data, which can be used to calibrate the model, and the other compartments have no applicable true data. Under the lockdown strategy, we assumed a constant population size (N) = 10,800,000. The modified COVID-19 epidemiological model can be expressed by the following nonlinear system of ordinary differential equations:

$$\begin{array}{l} \frac{dS}{dt}=-\beta \cdot \left(1-\frac{I}{N}\right)\cdot \frac{I}{N}\cdot S\\ \frac{dE}{dt}=\beta \cdot \left(1-\frac{I}{N}\right)\cdot \frac{I}{N}\cdot S-\lambda \cdot E\\ \frac{dI}{dt}=\lambda \cdot E-\alpha \cdot I-\gamma \cdot I\\ \frac{dH}{dt}=\alpha \cdot I-\mu \cdot H\\ \frac{dR}{dt}=\gamma \cdot I+\mu \cdot H \end{array}$$

The system is denoted as SEIHR with the initial values $\left(S_{0},E_{0},I_{0},H_{0},R_{0}\right)$, satisfying the following conservation law of the population:

$$\begin{array}{cc} N & =S_{0}+E_{0}+I_{0}+H_{0}+R_{0}\\ & =S(t)+E(t)+H(t)+R(t) \end{array}$$

Here, *N* indicates the total population without considering the numbers of natural births and deaths, and the definitions of the other parameters are shown in Table 1. Our model has only five model parameters, which is less than the parameters of SAPHIRE [26].

Using a second generator approach [28,29], we can obtain the control basic reproduction number as follows:

$$R_{0}=\frac{\beta }{\alpha +\gamma }\frac{S_{0}}{N}\approx \frac{\beta }{\alpha +\gamma }$$

Based on the parameters’ probability distribution function (PDF), we could easily deduce the PDF of R_0_ with the means and variance.

## 3. Model Calibration 

### 3.1. Data

**Figure 2:**
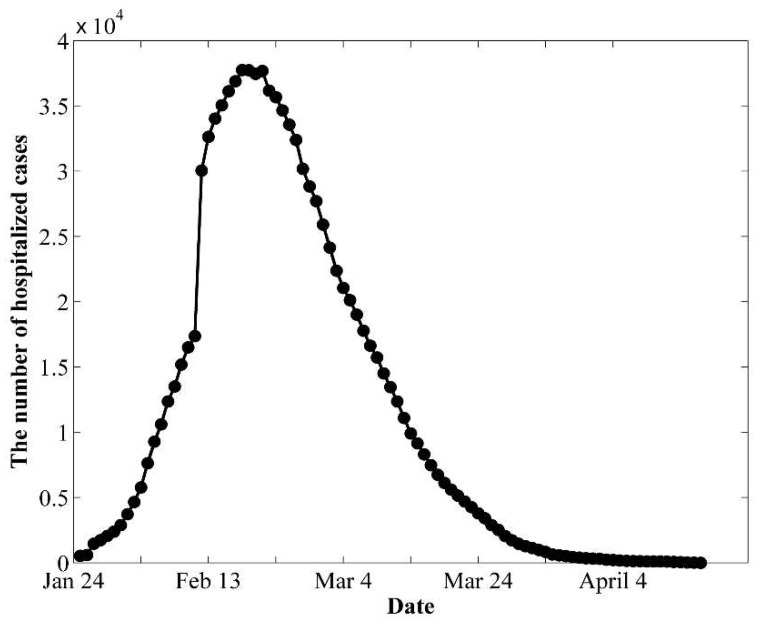
The public daily number of hospitalized cases of COVID-19 in Wuhan.

The observed data were collected from the official public data of the Wuhan Municipal Health Commission [30] and the big data of BAIDU [31]. To obtain reliable observed data, we used the numbers of daily confirmed cases (hospitalized cases), recovered cases, and deceased cases from 25 February to 26 April, after the lockdown of Wuhan city. There was a spike on 12 February, as shown in Figure 2. On 12 February, the clinical symptoms began to be used in Wuhan to confirm COVID-19 infection in addition to laboratory tests [30,32]. Therefore, we separated the study period into two stages (Figure 2), 25 January to 11 February (denoted as the 1st stage) and 12 February to 26 April (denoted as the 2nd stage), to investigate the epidemic trends before and after 12 February.

### 3.2. Calibration

There are generally two ways of calibrating the parameters: maximum likelihood and Bayesian analysis. For both approaches, the likelihood function is necessary. However, an appropriate likelihood function is usually difficult to obtain [33]. Approximate Bayesian computing (ABC) [34,35,36] is a set of methods that attempts to use the Bayesian idea without the likelihood function. In this study, we used ABC to estimate the parameters. Following the meaning of these parameters in Table 1, we can see that the other parameter is no larger than 1. Then, we assume that there is no further information for the priors of these parameters. As one of the most widely used noninformative priors, uniform PDF over the parameter spaces is chosen as the prior [37]. Analyzing the data, we observed that the parameter μ was no greater than 0.1, so we could assume that μ followed a uniform PDF at [0, 0.1], and all the other parameters were assumed to follow a uniform PDF at [0,1]. First, the parameters were sampled in uncertainty intervals 50,000 times with Latin hypercube sampling [38,39,40]. Then, we defined the following criteria for accepted samples:

$$\begin{array}{cc} d & =\sqrt{\frac{\sum_{t=1}^{Days}\left({\left(\ln(H(t))-\ln(H_{obs}(t)\right)}^{2}\right)}{Days}}\\ & \leq \varepsilon \sqrt{\frac{\sum_{t=1}^{Days}\left({\left(\ln(H_{obs}(t)\right)}^{2}\right)}{Days}} \end{array}$$

where ε is a small positive number. Here we take ε as 10^−6^.

After we obtained the accepted samples, the corresponding statistical values were deduced. For every sample of the parameters, we used MATLAB function to directly solve the ordinary differential system of the SEIHR model (see Appendix A). 

**Figure 3:**
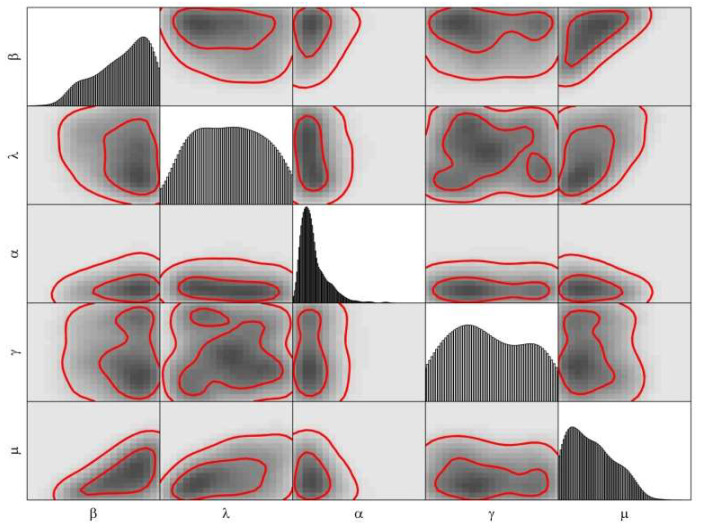
The joint posterior PDF of the parameters of the SEIHR model for the COVID-19 epidemic based on the public data for the 1st stage in Wuhan. The non-diagonal plots were the bivariate posterior PDF of the parameters.

**Figure 4:**
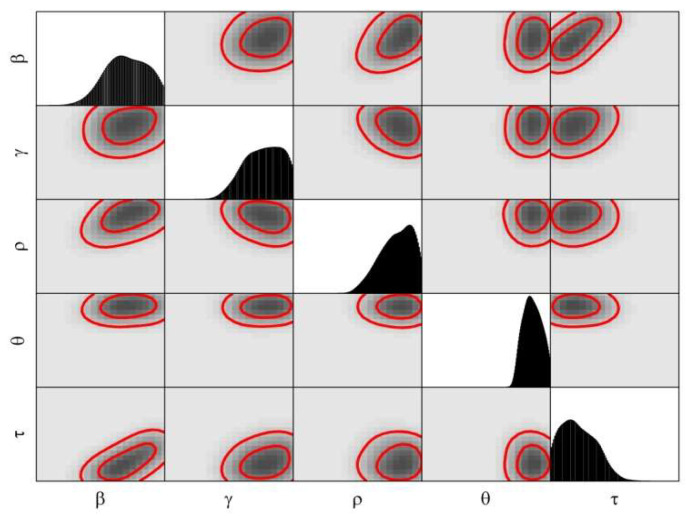
The joint posterior PDF of the parameters of the SEIHR model for the COVID-19 epidemic based on the public data for the 2nd stage of Wuhan. The non-diagonal plots were the bivariate posterior PDF of the parameters.

Figure 3 and Figure 4 are the posterior of model parameters for the COVID-19 epidemic in the 1st and 2nd stages of Wuhan.

## 4. Modeling the COVID-19 Epidemic in Wuhan

This section focuses on the spread of the COVID-19 epidemic in Wuhan after the lockdown of this city. We were mainly concerned with the number of hospitalized individuals with infections, which is the number of patients with confirmed infections, excluding those who had recovered or died.

**Figure 5:**
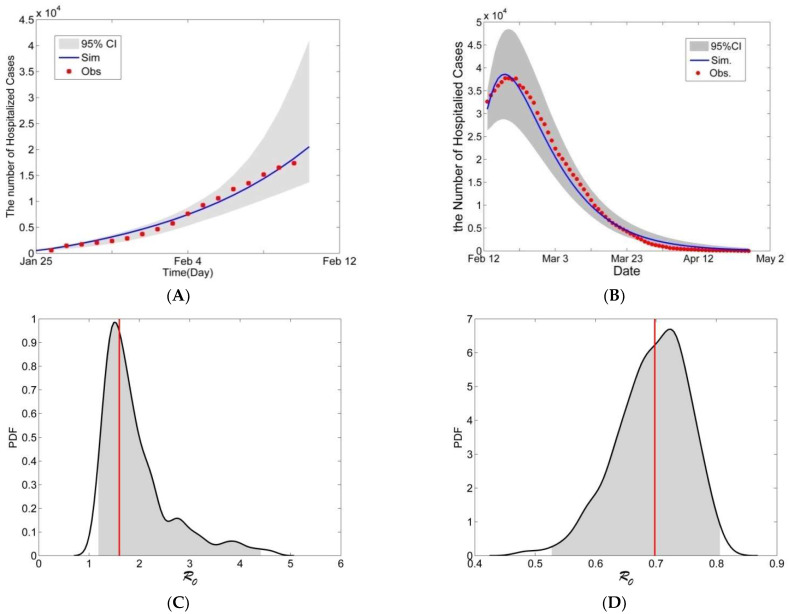
The model results for hospitalized infectious individuals during the two stages in Wuhan. (**A**,**B**) The gray region is the 95% confidence interval (CI) of the number of hospitalized infectious individuals. The blue line is the modeling result with the means of the posterior PDF of the parameters. (**C**,**D**) The gray regions are the 95% CI, the black lines are the probability density functions (PDFs), and the red lines are the mean R_0_ values.

We set the modeling result of 12 February with the means of the parameters for the 1st stage as the initial values of the model in the 2nd stage, except that the number of hospitalized infections was adjusted based on the public data for 12 February and the number of infections was also adjusted. According to the qualitative assessment, our model fit the case-incidence data well (Figure 5A for the 1st stage and Figure 5B for the 2nd stage in Wuhan). In those two stages, the 95% CIs of the hospitalized cases (the gray regions in Figure 5A,B) include the public data, and the modeling results with the corresponding means of the parameters are coherent with the observed data. We then estimated that the mean R_0_ values were 1.5517 (95% CI [1.1716, 4.4283]) for the 1st stage (Figure 5C) and 0.6937 (95% CI [0.5249, 0.8087]) for the 2nd stage in Wuhan (Figure 5D). These results indicate that R_0_ dropped below the self-sustaining threshold of 1 on 12 February 2020 in Wuhan; thus, that was the date on which control of the Wuhan epidemic was achieved, in as much as the infection rate was declining. We noticed that 12 February was the date that clinical symptoms began to be used to confirm COVID-19 in Wuhan. Figure 5A,B shows that our modeling result fits the observed data well.

**Figure 6:**
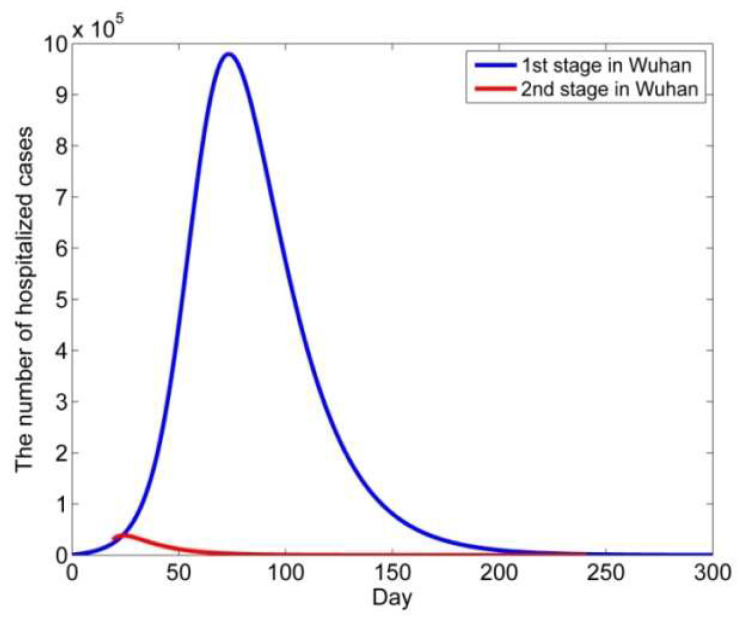
Modeling of the transmission dynamics of the epidemic: the model of the long-term dynamics in the two stages in Wuhan. The zero-day in the figure was 25 January 2020.

The public data for the COVID-19 epidemic in Wuhan shows that the peak number of hospitalized cases was 37,755 on 18 February 2020. However, supposing that no clinical symptoms were used to confirm the diagnosis of COVID-19 and performed a long-term simulation with the means of the parameters from the 1st stage in Wuhan, we estimated that the peak number of hospitalized cases would be reached on the 74th day after 25 January 2020, that is, on 8 April. The peak number of hospitalized cases was 979,966. The peak number of hospitalized patients was reduced by 96% (Figure 6).

**Figure 7:**
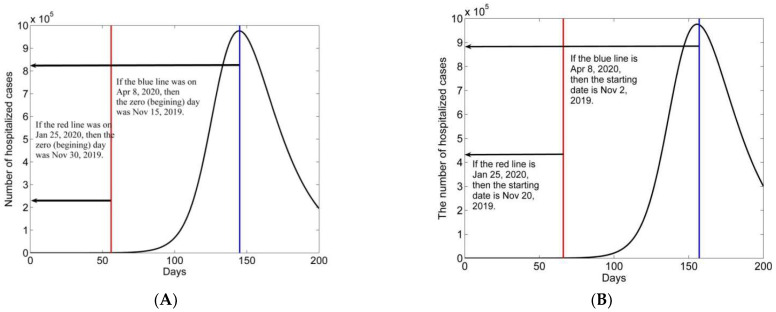
The long-term transmission dynamics of the model in the 1st stage in Wuhan with different initial values: (**A**) $\left(E_{0},I_{0},\;H_{0},\;R_{0})=(2,1,0,0\right)$; (**B**) $\left(E_{0},I_{0},H_{0},R_{0})=(1,0,0,0\right)$.

Furthermore, assuming that at the start of the COVID-19 epidemic, there were two latent, one infectious, and zero hospitalized cases, we modeled the long-term transmission of the COVID-19 epidemic. The result shows that the peak in hospitalized cases would have occurred on the 145th day, and the peak number would have been 976,472 (Figure 7A). The above two peak numbers are almost the same. The date that was closest to matching the public data on 25 January 2020, was the 56th day after the start of the COVID-19 epidemic. The modeling number of hospitalized infections was 560 (the number in the public data was 533) at the red line in Figure 7A. Furthermore, if we suppose that on the start date, the initial exposed class had only one case and that the infectious, hospitalized, and removed classes had zero cases, then the peak number of hospitalized cases would have occurred on the 157th day, and the date that was most similar to the public data for 25 January 2020 was the 66th day, on which there were 509 hospitalized cases (Figure 7B).

By performing long-term modeling of the 1st stage in Wuhan city, we estimated that the date with the peak number of hospitalized cases would have been 8 April, and the peak number of hospitalized cases would have been more than 900 thousand. If that had happened, the number of infected patients would have overwhelmed the public health services. The public data show that the peak number of hospitalized cases in the public data was 37,755 on 18 February 2020. Comparing the peak numbers in the public data and the above model, we see that the number was reduced by 96%, and the period was shortened by half. Regarding the long-term modeling with the calibrated parameters in the 1st stage, we used different initial values to estimate the start time. One estimated start date is 15 November 2019 and another estimated start date is 30 November 2019. 

## 5. Conclusions

The Chinese strategy of using clinical symptoms in diagnosing COVID-19 shortened the COVID-19 epidemic. In other words, this strategy made it possible to identify infected individuals as soon as possible. The other strategy adopted by the Chinese government, which involved building the Leishenshan, Huoshenshan, and Fangcang hospitals and recruiting health care teams from other provinces in China, enabled those with confirmed infections to be hospitalized as soon as possible. The results show that on 12 February, this strategy was the key to controlling the COVID-19 epidemic in Wuhan. From a mathematical point of view, if a large number of infectious cases suddenly turned into hospitalized cases, the spread of the COVID-19 epidemic would change.

Our estimated start dates agree with the results shown in Table 2. Remarkably, the period between the two estimated dates is almost equal to the incubation period. The period between the two dates is also consistent with the preliminary estimate of the incubation period (mean 7.5 days and 95% CI 5.3 to 19). Based on further assumptions, the estimated starting date of the COVID-19 epidemic in Wuhan was between 2 November and 20 November 2019.

In this study, to understand the transmission dynamics of the COVID-19 epidemic in Wuhan and other countries, we quantified the effectiveness of COVID-19 epidemic control strategies in Wuhan. Using public data, we considered the period to be consisting of two stages in Wuhan. The model was calibrated with public data. The results show that 12 February is the date on which control over the epidemic in Wuhan was achieved.

Overall, in this paper, we propose a modified SEIR mathematical model for the COVID-19 epidemic in Wuhan that started in January 2020 and was under control in a short period. In order to describe the transmission dynamics of the COVID-19 epidemic, this modified model considers the movement of people among five states: susceptible (S), exposed (E), infected (I), hospitalized (H), and removed (R). The model parameters are calibrated by ABC. The uncertainty of the number of hospital cases was modeled based on the calibrated parameters, and the uncertainty of the control basic reproduction number was analyzed. The modified mathematical model illustrates the good fit between observed and simulated hospitalized cases. Furthermore, we inversely deduced that the earliest transmission of COVID-19 in Wuhan possibly began on 2 November 2019, which is in accurate agreement with some known results [1,27,41,42]. The numerical results of the proposed modified SEIR model are valid for the COVID-19 epidemic in Wuhan, China. The numerical results show that the proposed model is validated for the COVID-19 epidemic. This study used as little early information about COVID-19 as possible to study the dynamic of COVID-19 in Wuhan. Thus, we hope that this study can help humans to quickly understand newly arisen viruses in the future.

## Figures and Tables

**Table 1:** Parameter definitions of the SEIHR model.

Parameter	Definition
*β*(1-*I*/*N*)	Transmission rate per day
*λ*	Rate of progression to infectious state per day
*α*	Rate of progression from the infectious to the hospitalized state per day
*γ*	Rate of progression from the infectious state to the removed state per day
*μ*	Rate of progression from the hospitalized state to the removed state per day

**Table 2:** Estimation of the start date of the COVID-19 epidemic in Wuhan.

Start Date	Evidence	Source
8 December 2019	Clinical cases	N Engl Med J [1]
1 December 2019	Clinical cases	Lancet [41]
November 2019	Inferred from the above result	Science [42]
Mid-October and mid- November 2019	Simulation	Science [27]
15 November 2019 to 30 November 2019	$\mathrm{Modeling}\;\mathrm{with}\;(E_{0},I_{0},H_{0},R_{0})=(2,1,0,0)$	This study
2 November 2019 to 20 November 2019	$\mathrm{Modeling}\;\mathrm{with}\;(E_{0},I_{0},H_{0},R_{0})=(1,0,0,0)$	This study

## Data Availability

The data for the COVID-19 epidemic in Wuhan, collected in the current study, are publicly available and can be found on the official website of the Wuhan Municipal Health Commission [30] and the big data of BAIDU [31]. The data are included in Appendix A.

## Appendix A. MATLAB Codes

function paper_seihr()

% Public data of Wuhan City. obs_c: cumulative confirmation cases; obs_r: cumulative recovery cases; obs_d: cumulative

%death cases; obs_h: current hospitalized cases

%obs_h=obs_c-obs_r-obs_d

obs_c=[618   698    1590    1905    2261    2639    3215    4109    5142    6384 ...

    8351  10117  11618  13603  14982  16902  18454  19558  32994  35991 ...

  37914  39462  41152  42752  44412  45027  45346  45660  46201  46607 ...

  47071  47441  47824  48137  48557  49122  49315  49426  49540  49671 ...

  49797  49871  49912  49948  49965  49978  49986  49991  49995  49999 ...

  50003];

 %%%---------------------------------------------------------------------

obs_r=[ 40     42     45        71        78        99        135      167       220      299 ...

      364    427    530      694      873     1040     1202    1373     1911    2281 ...

    2767   3180   3723    4484    5160    5713    6214     6206     8171   8946 ...

  10377  11793  13328  15826  17562  19227  21185   23031  24890 26316 ...

  27354  28511  29770  30933  31829  33041  34094   35197  36451 37632 ...

  38384];

 %%%---------------------------------------------------------------------

obs_d=[ 45     63       85      104     129     159     192     224     265     313 ...

       362    414     478     545     608     681     748     820    1036   1080 ...

   1123   1233   1309   1381   1497   1585   1684   1774   1856   1987 ...

   2043   2085   2104   2132   2169   2195   2227   2251   2282   2305 ...

   2328   2349   2370   2388   2404   2423   2430   2436   2446   2456 ...

   2469];

% the 1st stage from Jan 25 to Feb 11, 2020

obs_h_1=obs_c(1:18)-obs_r(1:18)-obs_d(1:18);

% the 2nd stage from Feb 12 to March 15, 2020

obs_h_2=obs_c(19:end)-obs_r(19:end)-obs_d(19:end);

N=10800000;

beta=0.61949;

lambda=0.48354;

alpha=0.16035;

gamma=0.23889;

mu=0.04284;

y0 = [N-4436-1914-618-40-45, 4436, 1914,618-40-45, 40+45];

ts=[0:1:200];

[t1, y1] = ode45(@odeseihr, ts,y0, [], beta,lambda,alpha,gamma,mu,N);

y0=[N-2-1,2,1,0,0];

[t1, y11] = ode45(@odeseihr, ts,y0, [], beta,lambda,alpha,gamma,mu,N);

y0=[N-1,1,0,0,0];

[t1, y12] = ode45(@odeseihr, ts,y0, [], beta,lambda,alpha,gamma,mu,N);

y0=y1(19,:);

y0(3)=y0(3)-(obs_c(19)-obs_r(19)-obs_d(19)-y0(4));

y0(4)=obs_c(19)-obs_r(19)-obs_d(19);

beta=0.50219;

lambda=0.42043;

alpha=0.56571;

gamma=0.49418;

mu=0.060524;

[t2, y2] = ode45(@odeseihr, ts,y0, [], beta,lambda,alpha,gamma,mu,N);

% the public data of the hospitalized cases in Wuhan city

figure(1);

plot(1:51,obs_c(1:51)-obs_r(1:51)-obs_d(1:51),'-o','markerFaceColor','b')

xlim([0 55])

ylabel('The number of hospitalized cases');

xlabel('Date');

set(gca,'XTickLabel',{'Jan 24','','Feb 3','','Feb 13','','Feb 23','','Mar 4','','Mar 14',''});

% The 1st stage from Jan 12 to Feb 11, 2020

figure(2);

plot(t1(1:18),y1(1:18,4),'LineWidth',2);

hold on

plot(0:17,obs_h_1,'o','MarkerFaceColor','b')

ylabel('The number of hospitalized cases');

xlabel('Date');

set(gca,'XTickLabel',{'Jan 25','','','','','Feb 4','','','','Feb 12'});

% The 2nd stage from Jan 12 to Mar 10, 2020

figure(3);

plot(t2(1:80),y2(1:80,4),'LineWidth',2);

hold on

plot(0:27,obs_h_2(1:28),'o','MarkerFaceColor','b');

ylabel('The number of hospitalized cases');

xlabel('Date');

set(gca,'XTickLabel',{'Feb 12','','Mar 3','','Mar 23','','Apr 12','','May 2'});

%the long period modeling of these two stages

figure(4)

plot(t1(1:150),y1(1:150,4),'LineWidth',2);

hold on

plot(t2(1:132)+18,y2(1:132,4),'r','LineWidth',2);

ylabel('The number of hospitalized cases');

xlabel('Day')

%the long period modeling of the 1st stage with different initial condition

figure(5)

plot(t1,y11(:,4),'LineWidth',2)

ylabel('The number of hospitalized cases');

xlabel('Day')

%the long period modeling of the 1st stage with different initial condition

figure(6)

plot(t1,y12(:,4),'LineWidth',2)

ylabel('The number of hospitalized cases');

xlabel('Day')

end

function dy = odeseihr(t, y,beta,lambda,alpha,gamma,mu, N)

%SEIHR ordinary differential system

dy = [ -beta*(1-y(3)/N)*y(1)*y(3)/N;

   beta*(1-y(3)/N)*y(1)*y(3)/N-lambda*y(2);

   lambda*y(2)-gamma*y(3)-alpha*y(3);

   alpha*y(3)-mu*y(4);

   gamma*y(3)+mu*y(4)];

end
